# Will the Effects of Sea-Level Rise Create Ecological Traps for Pacific Island Seabirds?

**DOI:** 10.1371/journal.pone.0136773

**Published:** 2015-09-23

**Authors:** Michelle H. Reynolds, Karen N. Courtot, Paul Berkowitz, Curt D. Storlazzi, Janet Moore, Elizabeth Flint

**Affiliations:** 1 U.S. Geological Survey, Pacific Island Ecosystems Research Center, PO Box 44, Hawai‘i National Park, Hawai‘i, United States of America; 2 Hawai‘i Cooperative Studies Unit, University of Hawai‘i at Hilo, Hawai‘i National Park, Hawai‘i, United States of America; 3 U.S. Geological Survey, Pacific Coastal and Marine Science Center, 400 Natural Bridges Drive, Santa Cruz, California, United States of America; 4 Saint Mary’s University, Halifax, Nova Scotia, Canada; 5 U.S. Fish and Wildlife Service, Pacific Islands Refuges and Monuments Office, 300 Ala Moana Blvd. Suite 5–231, Honolulu, Hawai‘i, United States of America; Behavioural Ecology & Ecophysiology group, GERMANY

## Abstract

More than 18 million seabirds nest on 58 Pacific islands protected within vast U.S. Marine National Monuments (1.9 million km^2^). However, most of these seabird colonies are on low-elevation islands and sea-level rise (SLR) and accompanying high-water perturbations are predicted to escalate with climate change. To understand how SLR may impact protected islands and insular biodiversity, we modeled inundation and wave-driven flooding of a globally important seabird rookery in the subtropical Pacific. We acquired new high-resolution Digital Elevation Models (DEMs) and used the Delft3D wave model and ArcGIS to model wave heights and inundation for a range of SLR scenarios (+0.5, +1.0, +1.5, and +2.0 m) at Midway Atoll. Next, we classified vegetation to delineate habitat exposure to inundation and identified how breeding phenology, colony synchrony, and life history traits affect species-specific sensitivity. We identified 3 of 13 species as highly vulnerable to SLR in the Hawaiian Islands and quantified their atoll-wide distribution (Laysan albatross, *Phoebastria immutabilis*; black-footed albatross, *P*. *nigripes*; and Bonin petrel, *Pterodroma hypoleuca*). Our models of wave-driven flooding forecast nest losses up to 10% greater than passive inundation models at +1.0 m SLR. At projections of + 2.0 m SLR, approximately 60% of albatross and 44% of Bonin petrel nests were overwashed displacing more than 616,400 breeding albatrosses and petrels. Habitat loss due to passive SLR may decrease the carrying capacity of some islands to support seabird colonies, while sudden high-water events directly reduce survival and reproduction. This is the first study to simulate wave-driven flooding and the combined impacts of SLR, groundwater rise, and storm waves on seabird colonies. Our results highlight the need for early climate change planning and restoration of higher elevation seabird refugia to prevent low-lying protected islands from becoming ecological traps in the face of rising sea levels.

## Introduction

The potential impacts of rapid climate change on tropical island ecosystems include changes in rainfall, wind patterns, storm frequency, coral bleaching, ocean acidity, sea-surface temperatures, disease incidence, and rising sea levels [1–6]. Satellite observations from 1993 to 2013 show global sea-level rise (SLR) to date meets predictions by the Intergovernmental Panel on Climate Change Assessment [7, 8]. Estimates including both ice melting and thermal expansion predict a potential global rise in sea level on the order of 1 m above the year 2000 reference level, by 2100 [8–15]; more extreme projections (> 1.5 m) depend on accelerated ice flow dynamics or high levels of greenhouse gas emissions [16–18]. Regardless, atmospheric warming and SLR are likely to continue due to system inertia and the amount of CO_2_ already present in the atmosphere [9, 19, 20].

Rising sea level is recognized as a major threat to coastal ecosystems and insular biodiversity [21–25]. Global SLR results in passive inundation, as well as increases in wave energy at shorelines, increasing wave run-up (i.e., the vertical extent of wave surge), coastal inundation, and erosion [26]. In our study area, the central North Pacific, storms annually generate wave heights in excess of 5 m [27]. Estimates of habitat loss from storm-driven flooding vary with topography, bathymetry, groundwater levels, and distribution of wildlife relative to inundation patterns [28]. Although islands are likely to be affected by numerous interrelated processes associated with SLR including erosion, accretion, and saltwater intrusion [29–31], we focus on storm-wave run-up and groundwater effects on insular nesting seabirds.

More than 18 million colonial seabirds nest on 58 islands protected within vast U.S. Marine National Monuments of the Pacific [32–37], and although seabirds have protection with the Migratory Bird Treaty Act, islands currently providing colonial seabirds refugia are at high risk from sudden wave-driven flooding events. Seabird colonies were extirpated from most Pacific islands after human colonization, invasion of mammalian predators, and other anthropogenic changes [38–41]. Remaining seabird nesting distributions include high-density colonies (e.g., 3 birds/m^2^, sooty terns (*Onychoprion fuscata*) [42]) and are often restricted to islands that are typically remote, predator-free, far from human settlements [39, 40].

Vulnerability of seabirds to climate change depends on a species’ exposure, sensitivity to stressors, and life history traits [43–45]. Some species are highly seasonal and synchronous breeders, while others have multiple or extended (asynchronous or aseasonal) breeding and exhibit variation in breeding phenology across their global range [46, 47]. Thus, catastrophic exposure to natural or anthropogenic perturbations (e.g., tsunamis, storms, oil spills) will vary according to the timing of perturbations and species-specific breeding seasonality, synchrony, and habitat overlap with the disturbance. Resilience to catastrophes will vary with the species’ breeding plasticity, degree of philopatry (i.e., natal and nest-site fidelity), propensity to disperse, and the availability of suitable alternative habitat [45].

Under conditions of rapid environmental change and rising sea levels, the concentration and attraction of colonial seabirds to protected low-lying islands may result in high reproductive failure or mortality, essentially creating an ecological trap in the absence of alternative predator-free, high elevation nesting habitat [48, 49]. Some species currently concentrated on low-lying islands may adjust to increasing overwash events and habitat loss by laying replacement eggs, increasing clutch size, increasing nest density or dispersing to more suitable habitat [50]. For example, sooty terns can lay multiple replacement clutches [51, 52], king penguins (*Aptenodytes patagonicus*) can renest [53], Nazca boobies (*Sula Granti*) can increase clutch size [54], and Caspian terns (*Hydroprogne caspia*) can disperse and breed at an alternate colony in the same season they are displaced from their historical colony [55]. In contrast, petrels and albatrosses (Procellariiformes) are constrained energetically to a single attempt per breeding season and are strongly philopatric [56].

To aide natural resource managers facing conservation decisions and climate change uncertainty, we quantified the potential effects of SLR inundation and wave-driven flooding for a range of SLR scenarios (+0.5, +1.0, +1.5, and +2.0 m) at Midway Atoll National Wildlife Refuge (NWR). We determined potential inundation extent and habitat loss for 13 seabird species. In addition to predicting nesting habitat exposure to inundation, we described species sensitivity to SLR by examining life history traits and the temporal overlap of nesting phenology and seasonal storms. For the three most vulnerable species identified, we quantified the population-level effects of passive inundation and wave-driven flooding. This is the first ecological study to simulate SLR with storm waves and groundwater impacts to seabird populations.

## Methods

### Study area

**Fig 1:**
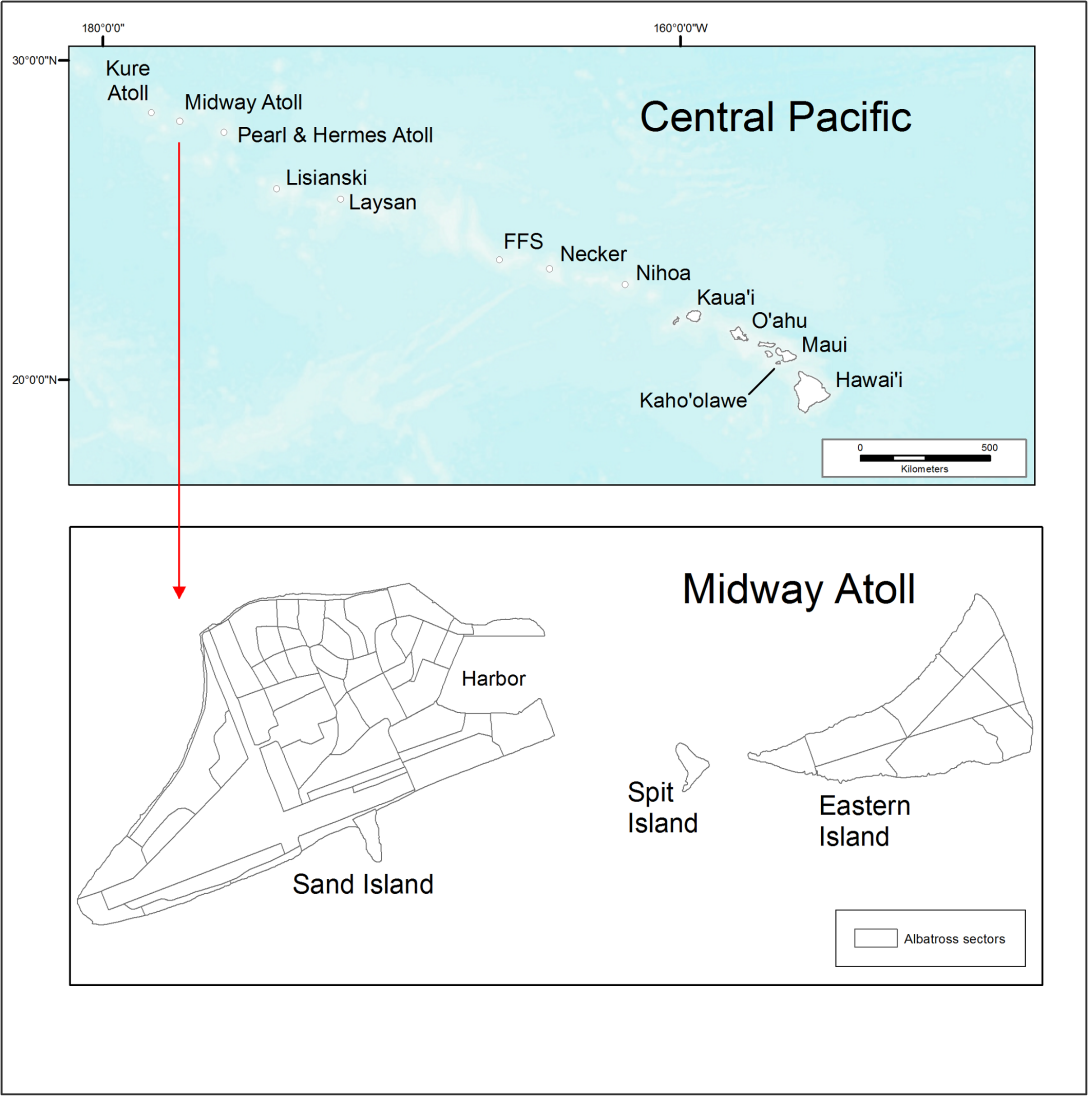
Map of the Hawaiian Islands in the Central Pacific Ocean, including Midway Atoll showing year 2011 albatross census sectors.

The Northwestern Hawaiian Islands (NWHI) are a World Heritage Site extending more than 1,400 km (Fig 1; [57]). The small islands were first protected as a bird reserve in 1909 [58] followed by the creation of national wildlife refuges [59] and the Papahānaumokuākea Marine National Monument, which includes 362,072 km^2^ of oceanic and island habitat [33, 60].

Midway Atoll NWR (28°11'41"–28°16'50" N and 177°18'38"–177°25'38" W) is near a region of the Pacific where high rates of local SLR (>5 mm/year) have been observed [7, 61]. The atoll consists of three islands: Sand (460.0 ha; mean elevation 3.2 m), Spit (5.8 ha; mean elevation 1.5 m), and Eastern (138.4 ha; mean elevation 2.6 m; Fig 1). In 1997, eradication of predatory introduced black rats (*Rattus rattus*) [62] allowed declining seabird populations to recover [39]. Currently, Midway Atoll is a breeding site of 19 seabird species, the endangered Hawaiian monk seal (*Monaschus schauinslandi*), and a reintroduced population of endangered Laysan teal (*Anas laysanensis*). Nearly 75% of the global breeding population of Laysan albatrosses and one-third of black-footed albatrosses nest at Midway Atoll; it is also home to one of the world’s largest Bonin petrel colonies (Table 1; [63, 64]).

**Table 1:** Conservation status (2015) and population status of albatrosses (*Phoebastria immutabilis* and *P*. *nigripes*) and Bonin petrels (*Pterodroma hypoleuca*) in the Northwestern Hawaiian Islands (NWHI). Compiled from the International Union for Conservation of Nature [65], the US Fish and Wildlife Service (USFWS) Endangered Species Program [66] and its list of Birds of Conservation Concern (BCC; [67]), and the State of Hawai‘i, Department of Land and Natural Resources [68].

	Status			Population		
Common name	IUCN	U.S. Federal	State of Hawai‘i	NWHI breeding pairs reported	Proportion of global breeding population restricted to NWHI	Proportion of global breeding population on Midway Atoll
Laysan albatross	Near Threatened	BCC		590,000^a^	> 0.95^a^	c. 0.75
Black-footed albatross	Near Threatened	BCC	Threatened	60,000^a^	> 0.95^a^	c. 0.36
Bonin petrel	Least Concern			> 396,000^b^	> 0.95^c^	> 0.20

### Digital elevation and inundation modeling

To model SLR inundation, we created a seamless topographic-bathymetric digital elevation model (DEM) based on a one-third arc second (approximately 10 m resolution) bathymetric grid [69] and a 1-m topographic DEM [70]. We used the bathymetric data [71] to model wave heights at the shoreline and the topographic data [70] to estimate the extent of inundation [23, 72]. Next, within a GIS framework (ArcGIS 10.0 [73]) we mapped both passive and wave-driven flooding at Midway Atoll for four potential SLR scenarios (+0.5, +1.0, +1.5, and +2.0 m, relative to 2011 sea levels). The passive approach represents the amount of inundation due to SLR only (i.e., during calm conditions), while the wave-driven model considers the additive effect of storm events.

We used the Delft3D WAVE model [74, 75] and ArcGIS 10.0 [73] to compute and project wave-driven water levels up the beach face during storm events. We extracted the top 5% of winter storm waves from hourly US Army Corps of Engineers Wave Information System data (over 200,000 hindcast data points) for the years 1981–2004 [27, 76, 77] for a location 375 km east of Sand Island (28°00’ N, 174°00’ W). These data served as input parameters (wave height, period and direction) to the Delft3D WAVE module (SWAN, or Simulating Waves Nearshore) for predicting wave behavior [78, 79]. For calibration and validation of this wave model for the Northwestern Hawaiian Islands see [80, 81]. We conducted a sensitivity analysis for our wave run-up model by varying the input parameter values and evaluating the effect on wave run-up to variations in these parameters. We tested a range of errors for wave height from 0–6 m at 0.5 m increments, wave period from 0–14 s at 0.2 s increments, and beach slope from 1–6% at 1% increments. We used mean high water (MHW) as the vertical reference since this level generally represents a worst-case scenario (i.e., flooding coincident with mean high tide). MHW was delineated using satellite imagery (collected on 05 August 2011 [82]), verified tide (at Station ID 1619910 [83]), and tidal bench mark sheets [83].

Inundation levels in this study have inherent uncertainty in both the future rate of sea-level rise and the margin of error due to variation in tide, topographic elevations, and wave-driven water levels [72, 84, 85]. Since these margin of error terms (expressed as standard deviation [SD]) are uncorrelated, they can be combined into an overall uncertainty term [86]:

$$SD_{Total}=\;{\left(SD_{Tidal}{}^{2}+\;SD_{Topography}{}^{2}+\;SD_{Run-up}{}^{2}\right)}^{0.5}$$

We estimated the overall vertical root mean square error (RMSE) as 0.41 m [28, 72]. This RMSE has a variable effect on horizontal inundation extent, as areas with gradual slopes exhibit more horizontal uncertainty than areas with steep slopes. At each delineated boundary, a 50% probability of flooding exists; for elevations above the boundary, the probability decreases according to a normal probability distribution [28].

Hydrological findings on the main Hawaiian Islands [87–89], Laysan Island [90], and Midway Atoll [91] suggest that percolation of seawater into the water table may produce a rise in groundwater levels comparable to SLR, especially over long time periods. Therefore, we included the effects of groundwater by assuming that a rising water table will cause inundation to occur in all areas below a given SLR threshold. For a comparison of SLR models without ground water rise see [72].

### Seabird habitat and nest exposure

We quantified the potential losses of seabird habitat under a range of sea-level scenarios by overlaying inundation extents on land cover maps of Midway Atoll [82]. Land cover classes consisted of 9 categories, including four broad vegetation classes (tree/shrub, grass/herbaceous cover, vine/ground cover, and partially vegetated former runway), one individual tree species (ironwood or *Casuarina equisetifolia*), two unvegetated classes (beach subject to tidal and wave action; and bare ground inland of the beach zone), wetland areas, and human structures (see [72] for detailed descriptions). Land cover classes used by nesting seabirds (Table 2) were identified to evaluate habitat flooding on 13 seabird species with populations greater than 100 nesting pairs.

**Table 2:** Types of nesting habitat used by seabirds and rankings of species’ sensitivity to storm-wave overwash at Midway Atoll. Seabird behavior and nesting habitat by land cover class identified from satellite imagery at Midway Atoll (2011). Species’ sensitivity to population-level effects of storm-wave overwash were ranked from least (1) to most (3) sensitive. Nesting philopatry (natal and nest site fidelity) of species were ranked relatively as low (1), moderate (2), or high (3) and nesting frequency as annual–semi-annual (1), annual (2), annual–alternate (3; see [28] for additional details). Land cover classes beach, wetland, and infrastructure are not used as seabird nesting habitat at Midway Atoll (see [23]). Species in **bold** lay at most a single egg per year. Species with populations less than 100 nesting pairs at Midway Atoll were excluded.

	Life history		Land cover class					
Common name	Nesting philopatry	Nesting frequency	Tree/ shrub	*Casuarina equisetifolia*	Grass/ herbaceous cover	Vine/ ground cover	Partially vegetated former runway	Bare ground
Tree- or shrub-nesting								
Red-footed booby	2	2	**√**	**√**				
Great frigatebird	1	3	**√**					
Black noddy	2	1	**√**	**√**				
White tern	2	1	**√**	**√**				
Ground-nesting								
**Black-footed albatross**	3	3		**√**	**√**	**√**	**√**	**√**
**Laysan albatross**	3	3		**√**	**√**	**√**	**√**	**√**
**Christmas shearwater**	3	3	**√**	**√**	**√**	**√**	**√**	
Red-tailed tropicbird	3	3	**√**	**√**	**√**			
Gray-backed tern	1	2				**√**	**√**	**√**
Sooty tern	1	2			**√**	**√**	**√**	**√**
Brown noddy	1	2	**√**	**√**	**√**	**√**	**√**	**√**
Burrow-nesting								
**Bonin petrel**	3	2	**√**	**√**	**√**	**√**		
**Wedge-tailed shearwater**	3	3	**√**	**√**	**√**	**√**		**√**

Additionally, to assess SLR vulnerability, we evaluated the chronological exposure of seabird’s breeding phenology with historical storm waves. We also ranked species sensitivity to SLR from least (1) to most (3) susceptible to effects of storm-wave overwash by evaluating species life history traits. Relative nesting philopatry (natal and nest-site fidelity) was categorized as low (1), moderate (2), or high (3) and nesting frequency as annual–bi-annual (1), annual (2), or annual–alternate (3). For the three species that we identified to be most vulnerable to SLR and seasonal storm effects, we quantified potential population-level effects of inundation at Midway Atoll (see methods below).**Distribution and abundance of nests**

For the albatrosses and Bonin petrels, we collected nest location data and created contemporary maps of breeding distribution and abundance across Midway Atoll to spatially delineate and quantify areas of exposure to SLR scenarios.

#### Albatrosses

Laysan albatrosses construct simple ground nests of sand and debris, and nest throughout Midway Atoll, whereas black-footed albatrosses typically construct ground nests in open habitat primarily near the coast ([92–95]; also see Results). Egg-laying begins in November and chicks begin to fledge in July [92].

We participated in an atoll-wide census of albatross nests at Midway from 16 December 2011 to 8 January 2012. The atoll was divided into historically established census sectors (Fig 1; *n* = 61 sectors; mean = 9.3 ha/sector, range 1.4–98.8 ha [including non-habitat]). We georeferenced sector boundaries to WorldView-2 satellite imagery (collected 05 Aug 2011; [82]). For coastal sectors, the seaward extent of suitable nesting habitat was delineated based on 2012 black-footed albatross nest sites (see below), elevation contours (USGS data), and satellite imagery [82]. Surveyors walked parallel transects approximately 3 m apart counting every nest. We calculated nest density (nests/ha) for each sector based on suitable nesting habitat only (e.g., excluding infrastructure and wetlands).

We collected black-footed albatross nest location data using hand-held global positioning system (GPS) units (Trimble GeoXM and GeoXT unit; ± < 3 m accuracy). During the atoll-wide census, every nest was counted; however, time constraints prevented collection of GPS location data of every nest. We collected GPS locations for nearly 70% (419.7 ha) of the atoll area, prioritizing coastal high density nesting areas. In interior areas without GPS location data, we assumed a uniform distribution of nests across the suitable habitat within each census sector (Fig 1).

#### Bonin Petrels

Bonin petrels dig tunnels and nest in underground burrows [96]. Egg laying begins in mid-January and chicks begin to fledge in mid-May [97]. We delineated all Bonin petrel nesting areas on Sand and Eastern islands during 29 January–7 February 2008 using a hand-held GPS (Garmin GPSMAP 60CSx, ± < 10 m accuracy) and estimated the breeding population’s abundance on Sand Island. Detailed methods are described by Moore [98]. Briefly, we designated low, medium, or high density colony areas based on the distances between occupied burrows. Average nest density (low = 386 nests/ha, medium = 709 nests/ha, and high = 1329 nests/ha) was used to estimate breeder abundance in each nesting area on Sand Island.

To quantify nest inundation under a range of sea-level scenarios and modeling approaches, we overlaid inundation and flooding extent with our maps of breeding distribution and abundance for albatrosses in 2011–2012 and Bonin petrels in 2008 (Fig 1). We assumed that inundation would typically result in a loss of nest habitat, eggs, or chicks.

## Results

### Inundation of seabird habitat

Less than 10% of the atoll’s land area was affected at +1.0 m SLR with passive and wave-driven projections (Table 3). When SLR reached +2.0 m SLR, 19% (114.3 ha) of the atoll was inundated under the passive modeling approach and 55% (330.8 ha) flooded when storm waves were included (Table 3).

**Table 3:** The projected area inundated (in hectares as a proportion of land cover class, or total island area) at Midway Atoll for four scenarios of sea-level rise and two modeling approaches, passive (a) and wave-driven (b), with effects of rising groundwater.

**a**		**Passive Inundation**							
		SLR = +0.5 m		SLR = +1.0 m		SLR = +1.5 m		SLR = +2.0 m	
Land cover class	Current area (ha)	Area (ha)	Proportion	Area (ha)	Proportion	Area (ha)	Proportion	Area (ha)	Proportion
Tree/shrub	59.6	0.0	0.00	0.0	0.00	1.0	0.02	8.9	0.15
*Casuarina equisetifolia*	75.7	0.0	0.00	0.1	0.00	1.7	0.02	11.2	0.15
Grass/herbaceous cover	166.1	0.0	0.00	0.1	0.00	2.2	0.01	21.6	0.13
Vine/ground cover	60.3	0.0	0.00	0.1	0.00	0.5	0.01	9.0	0.15
Partially vegetated former runway	36.7	0.0	0.00	0.0	0.00	0.2	0.01	4.1	0.11
Bare ground	43.5	0.0	0.00	0.3	0.01	4.3	0.10	13.3	0.31
Beach	33.7	7.5	0.22	15.4	0.46	23.6	0.70	29.5	0.88
Wetland	2.1	0.0	0.00	0.3	0.14	1.5	0.71	1.9	0.90
Infrastructure	126.5	0.5	0.00	0.7	0.01	1.2	0.01	14.8	0.12
Total atoll area	604.2	8.0	0.01	17.0	0.03	36.2	0.06	114.3	0.19
**b**		**Wave-driven Inundation**							
		SLR = +0.5 m		SLR = +1.0 m		SLR = +1.5 m		SLR = +2.0 m	
Land cover class	Current area (ha)	Area (ha)	Proportion	Area (ha)	Proportion	Area (ha)	Proportion	Area (ha)	Proportion
Tree/shrub	59.6	0.0	0.00	2.6	0.04	8.9	0.15	23.7	0.40
*Casuarina equisetifolia*	75.7	0.0	0.00	2.4	0.03	11.6	0.15	31.7	0.42
Grass/herbaceous cover	166.1	0.0	0.00	7.8	0.05	36.5	0.22	96.5	0.58
Vine/ground cover	60.3	0.0	0.00	4.0	0.07	14.1	0.23	33.6	0.56
Partially vegetated former runway	36.7	0.0	0.00	1.6	0.04	10.9	0.30	33.9	0.92
Bare ground	43.5	1.4	0.03	8.2	0.19	17.4	0.40	31.7	0.73
Beach	33.7	16.0	0.47	25.3	0.75	30.8	0.91	33.1	0.98
Wetland	2.1	0.0	0.00	0.3	0.14	1.4	0.67	1.9	0.90
Infrastructure	126.5	0.9	0.01	3.1	0.02	16.5	0.13	44.7	0.35
Total atoll area	604.2	18.3	0.03	55.3	0.09	148.1	0.25	330.8	0.55

Using satellite imagery we estimated grass/herbaceous cover (e.g., *Eragrostis variabilis*, *Verbesina encelioides*) comprised 27% (166.1 ha) of the atoll area, vine/ground cover (e.g., *Ipomoea pes-caprae*, *Tribulus cistoides*) 10% (60.3 ha), and bare ground 7% (43.5 ha); these land cover types provided nesting habitat for 9 species including albatrosses, shearwaters, and terns (Tables 2 and 3). Trees and shrubs (e.g., *Casuarina equisetifolia*, *Scaevola taccada*, *Tournefortia argentea*) covered 22% (135.3 ha) of the atoll and were used as nesting habitat by red-footed boobies (*Sula sula*), great frigatebirds (*Fregata minor*), black noddies (*Anous minutus*), and white terns (*Gygis alba*); additionally burrow-nesting Bonin petrels and wedge-tailed shearwaters (*Puffinus pacificus*), and ground-nesting Christmas shearwaters (*Puffinus nativitatus*), red-tailed tropicbirds (*Phaethon rubricauda*), and brown noddies (*Anous minutus*) nest underneath the trees and shrubs (Tables 2 and 3). Human infrastructure including buildings, roads, and tarmac made up 21% (126.5 ha) of the atoll (Table 3).

With the passive modeling approach, no more than 2% of any nesting habitat type was projected to be lost at or below +1.5 m SLR, except for bare ground which decreased by 10%. However, with the wave-driven models, 15–40% of all nesting habitat was lost at +1.5 m SLR. Loss of tree/shrub habitat was 15% for both the +1.5 m SLR scenario under the wave-driven flooding approach and the +2.0 m SLR scenario under the passive inundation approach (Table 3).

### Seasonal storms, breeding phenology, and life history traits

**Fig 2:**
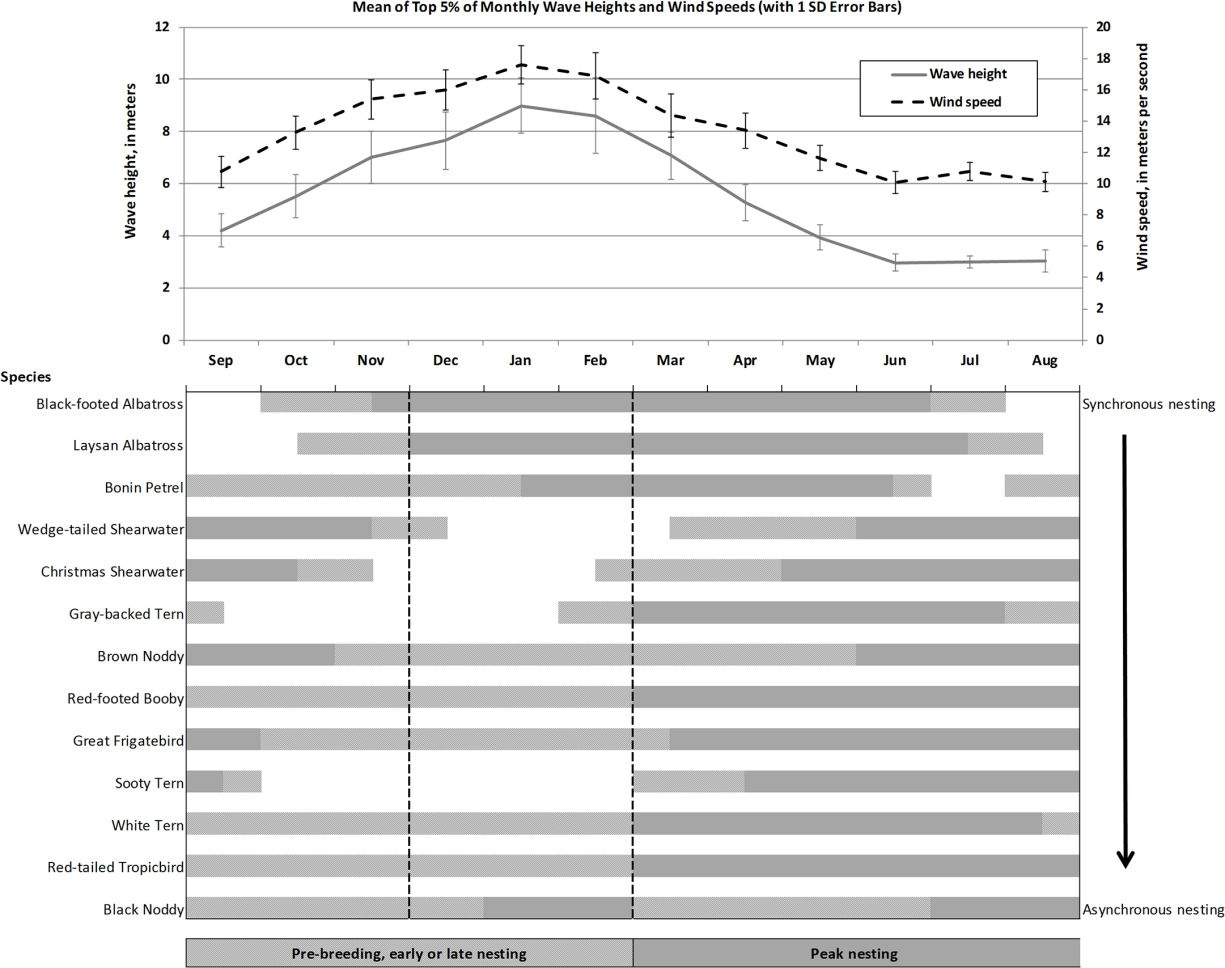
Breeding phenology and synchrony of select (> 100 pairs) seabird species at Midway Atoll compared with the monthly mean of the top 5% of wave heights and wind speeds. Monthly mean of top wave heights and wind speeds (± 1 standard deviation; [27]) were derived from hindcast data for the years 1981–2004 at 28° 00’ N, 174° 00’ W (375 km east of Sand Island). Peak nesting period includes incubation and chick rearing. Species listed in descending order of nesting colony synchrony (observed at Midway Atoll).

Hindcast data indicate that strong winds (max 17.6 m/s) and high surf (max 9.0 m) were most likely to occur during winter months (Dec–Feb; [23]). Overlap of breeding phenology, colony synchrony, and the expected period of highest wave energy and wind speed varied by species (Fig 2). Egg and chick incubation of albatrosses and Bonin petrels coincides with seasonally high wind and surf conditions (Fig 2). For aseasonal nesting populations such as black noddies and less synchronous nesters such as red-tailed tropicbirds and white terns, exposure of breeding colonies to storm-wave flooding and high winds is expected to have limited overlap temporally with reproductive effort (Fig 2). At Midway Atoll, gray-backed terns (*Onychoprion lunatus*) and great frigatebirds begin nesting after the typical storm season and there is little chronological overlap between the severe storm season and nesting of wedge-tailed shearwaters, Christmas shearwaters, or sooty terns (Fig 2). The relative ranking of species sensitivity to SLR (philopatry and renesting propensity) showed black noddies, white terns, and sooty terns having the lowest sensitivity (i.e., most flexibility), and the albatrosses, petrels, and shearwaters the least flexibility (Table 2).

#### Laysan Albatross

**Fig 3:**
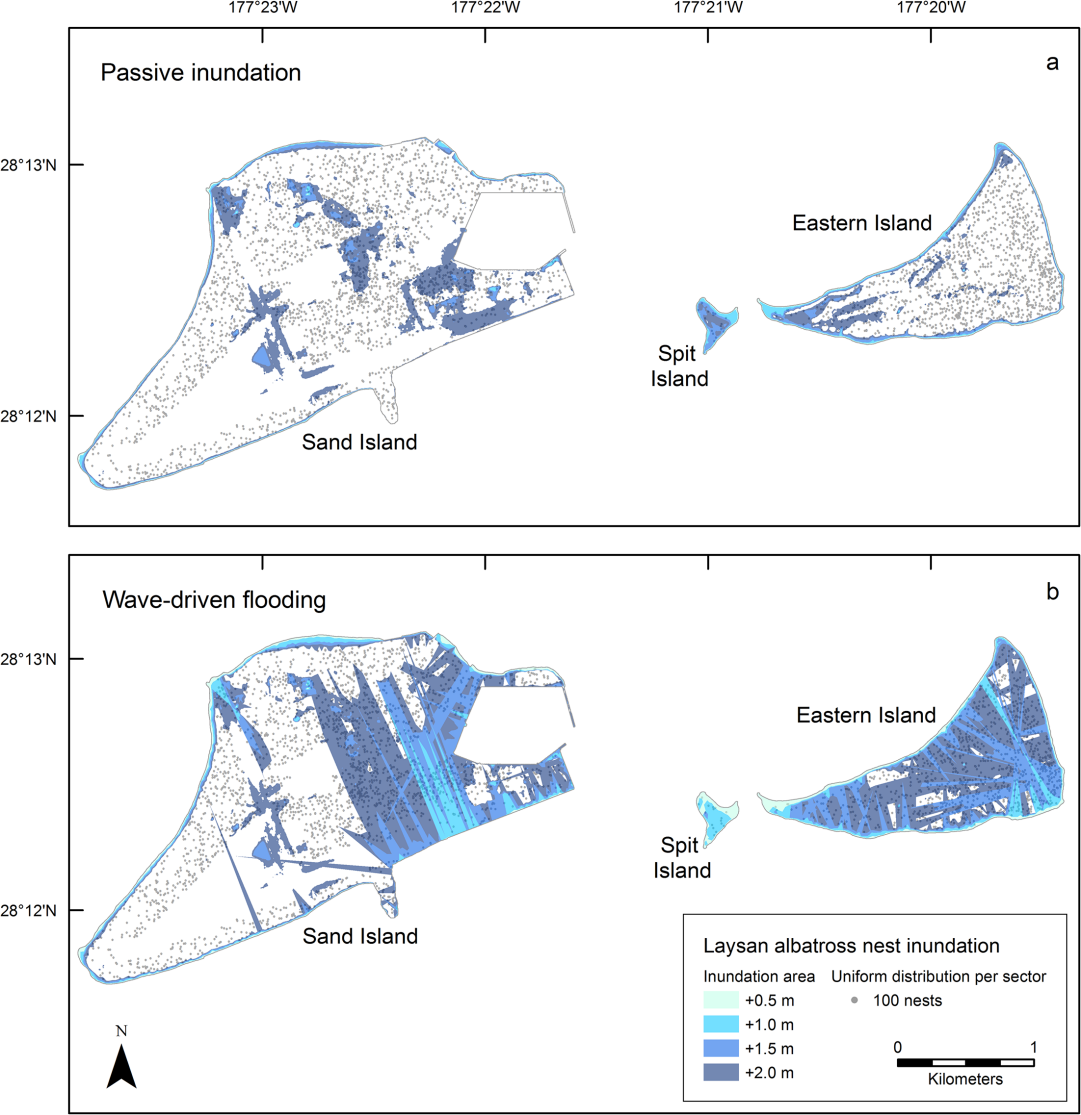
Projected inundation of Laysan albatross nests at Midway Atoll for four sea-level rise scenarios (+ 0.5, 1.0, 1.5, and 2.0 m) using passive (a) and wave-driven (b) models, including groundwater rise.

Laysan albatross nests at Midway Atoll totaled 388,017 at the peak of the 2011–2012 breeding season; 69% were on Sand Island (583 nests/ha), 31% on Eastern Island (856 nests/ha), and less than 1% on Spit Island (278 nests/ha). Laysan albatross nests occurred in all sectors of Midway Atoll and in all nesting habitat classes, except tree/shrub (Figs 1 and 3). Mean nest density among survey sectors was 1118 nests/ha of suitable habitat (range 386–2546, SD = 612 [excluding one outlier]).

Using the passive inundation approach, only 2% of Laysan albatross nests were projected to be inundated at +1.5 m SLR (Table 4). Once SLR reached +2.0 m, 15% of the nests atoll-wide experienced inundation, with 17% of the nests on Sand and 10% of the nests on Eastern islands flooded (Table 4). On Sand Island groundwater rise flooded two areas with high Laysan albatross densities (Fig 3).

**Table 4:** Projected Laysan albatross nest inundation at Midway Atoll for four sea-level rise scenarios and two inundation models, including groundwater rise. Nest inundation based on the 2011/2012 breeding season nest census at Sand (267,978 nests), Spit (1,615 nests), and Eastern (118,424 nests) islands, totalling 388,017 nests atoll-wide.

		SLR Scenarios							
		+0.5 m		+1.0 m		+1.5 m		+2.0 m	
Model	Island	No. nests	Proportion of nests	No. nests	Proportion of nests	No. nests	Proportion of nests	No. nests	Proportion of nests
Passive inundation	Sand	0	0.00	207	0.00	4,695	0.02	45,653	0.17
	Spit	0	0.00	37	0.02	1,016	0.63	1,611	1.00
	Eastern	0	0.00	101	0.00	1,683	0.01	12,124	0.10
	Atoll-wide	0	0.00	345	0.00	7,394	0.02	59,388	0.15
Wave-driven flooding	Sand	213	0.00	11,532	0.04	40,409	0.15	127,892	0.48
	Spit	356	0.22	1,564	0.97	1,615	1.00	1,615	1.00
	Eastern	266	0.00	9,452	0.08	48,700	0.41	106,949	0.90
	Atoll-wide	835	0.00	22,548	0.06	90,724	0.23	236,456	0.61

At the +0.5 m SLR scenario, the wave-driven and passive models estimate similar inundation extents. The proportion of inundated nests at +2.0 m passive SLR was similar to the proportion impacted by wave-driven flooding at +1.0 to +1.5 m SLR (Table 4). With waves at +1.0 m of SLR, nest flooding was less than 9% on Sand and Eastern Island; however, at +2.0 m SLR, 48% of the nests on Sand and 90% of nests on Eastern flooded (Table 4). At +1.5 and +2.0 m of SLR, wave-driven water flooded most of Eastern Island, and all of Spit Island (Fig 3).

### Black-footed Albatross

We counted 25,433 black-footed albatross nests: 59% on Sand Island (33 nests/ha), 41% on Eastern Island (75 nests/ha), and less than 1% on Spit Island (5 nests/ha). Averaged across the four SLR scenarios and modeling approaches, we collected GPS locations for black-footed albatross nests in more than 70% of suitable habitat predicted to flood.

**Fig 4:**
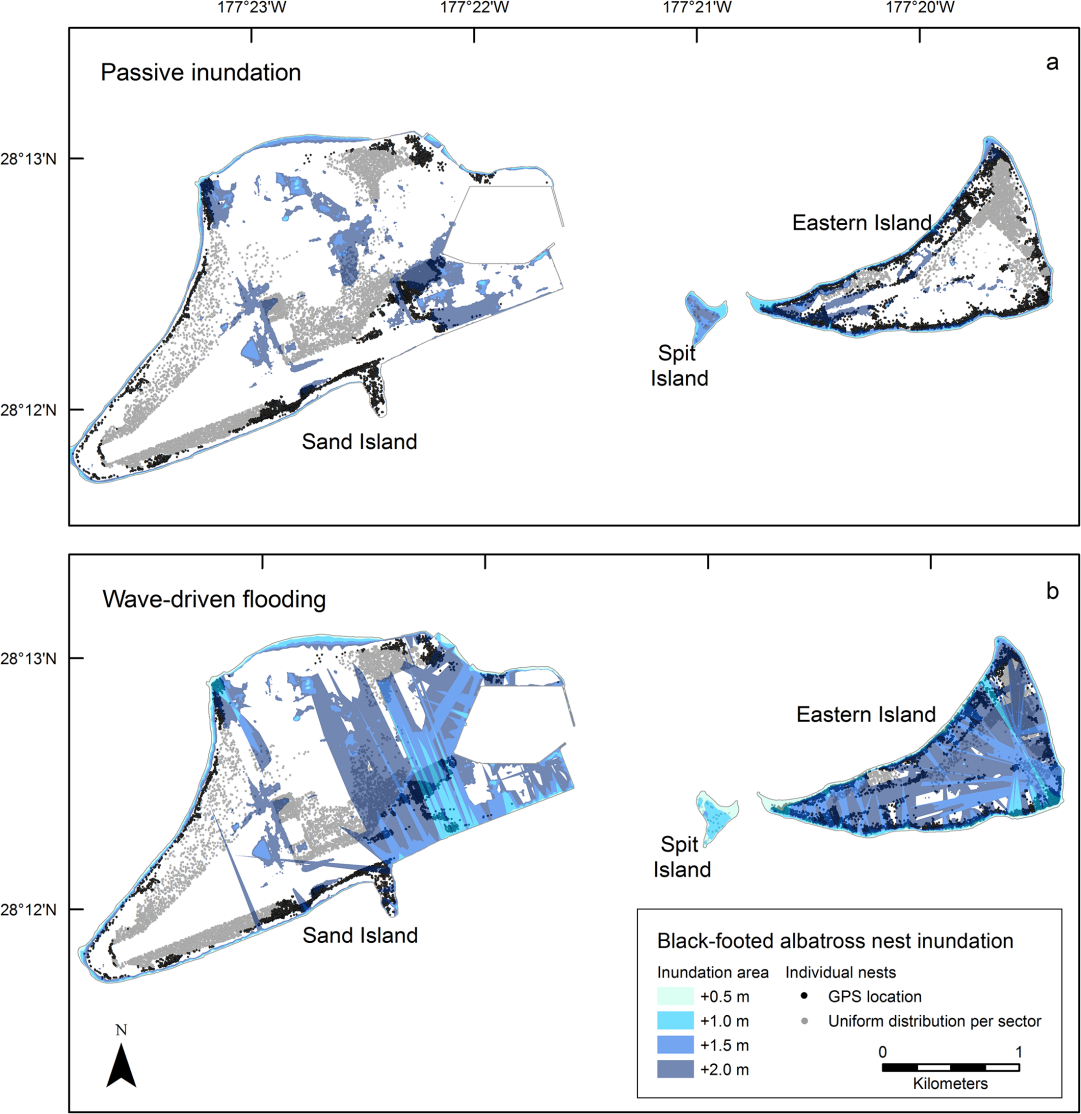
Projected inundation of black-footed albatross nests at Midway Atoll for four sea-level rise scenarios (+ 0.5, 1.0, 1.5, and 2.0 m) using passive (a) and wave-driven (b) models, including groundwater rise. Individual nest sites with GPS locations (± < 3 m accuracy) are indicated in black, approximate site locations of nests counted within census sectors are indicated in gray (assumes uniform distribution in suitable habitat [excludes infrastructure, shrubs and wetlands]).

We acquired GPS locations for 13,225 (52%) of the nests in the 2011–2012 breeding season. Black-footed albatross nests did not occur in 44% (*n* = 61) of interior sectors (i.e., those without coastline; Figs 1 and 4). Mean nest density among survey sectors was 66.3 nests/ha of suitable habitat (range 0–413, SD = 90.3).

Using the passive modeling approach, less than 5% of the nests at Sand and Eastern islands were inundated at any SLR scenario at or below +1.5 m (Table 5). Once SLR reached +2.0 m, 18% of black-footed albatross nests atoll-wide were inundated (Table 5). Passive coastal inundation coincided with high black-footed albatross densities in the northwest and southeast of Sand Island and in the north of Eastern Island (Fig 4). With wave-driven effects, 39 and 91% of nests at Sand and Eastern Islands, respectively, were flooded at +2.0 m SLR (Table 5). Wave-driven flooding of black-footed albatross nests occurred across all shorelines (Fig 4).

**Table 5:** Projected black-footed albatross nest inundation at Midway Atoll for four sea-level rise scenarios and two inundation models, including groundwater rise. Nest distribution and abundance based on the 2011/2012 breeding season nest census at Sand (15,002 nests), Spit (28 nests), and Eastern (10,413 nests) islands, totalling 25,443 atoll-wide.

		SLR Scenarios							
		+0.5 m		+1.0 m		+1.5 m		+2.0 m	
Model	Island	No. nests	Proportion of nests	No. nests	Proportion of nests	No. nests	Proportion of nests	No. nests	Proportion of nests
Passive inundation	Sand	0	0.00	0	0.00	27	0.00	2,456	0.16
	Spit	0	0.00	1	0.04	18	0.64	28	1.00
	Eastern	0	0.00	28	0.00	470	0.05	2,010	0.19
	Atoll-wide	0	0.00	29	0.00	515	0.02	4,494	0.18
Wave-driven flooding	Sand	1	0.00	995	0.07	2,375	0.16	5,799	0.39
	Spit	6	0.21	27	0.96	28	1.00	28	1.00
	Eastern	142	0.01	1,534	0.15	4,769	0.46	9,455	0.91
	Atoll-wide	149	0.01	2,556	0.10	7,172	0.28	15,282	0.60

### Bonin Petrel

**Fig 5:**
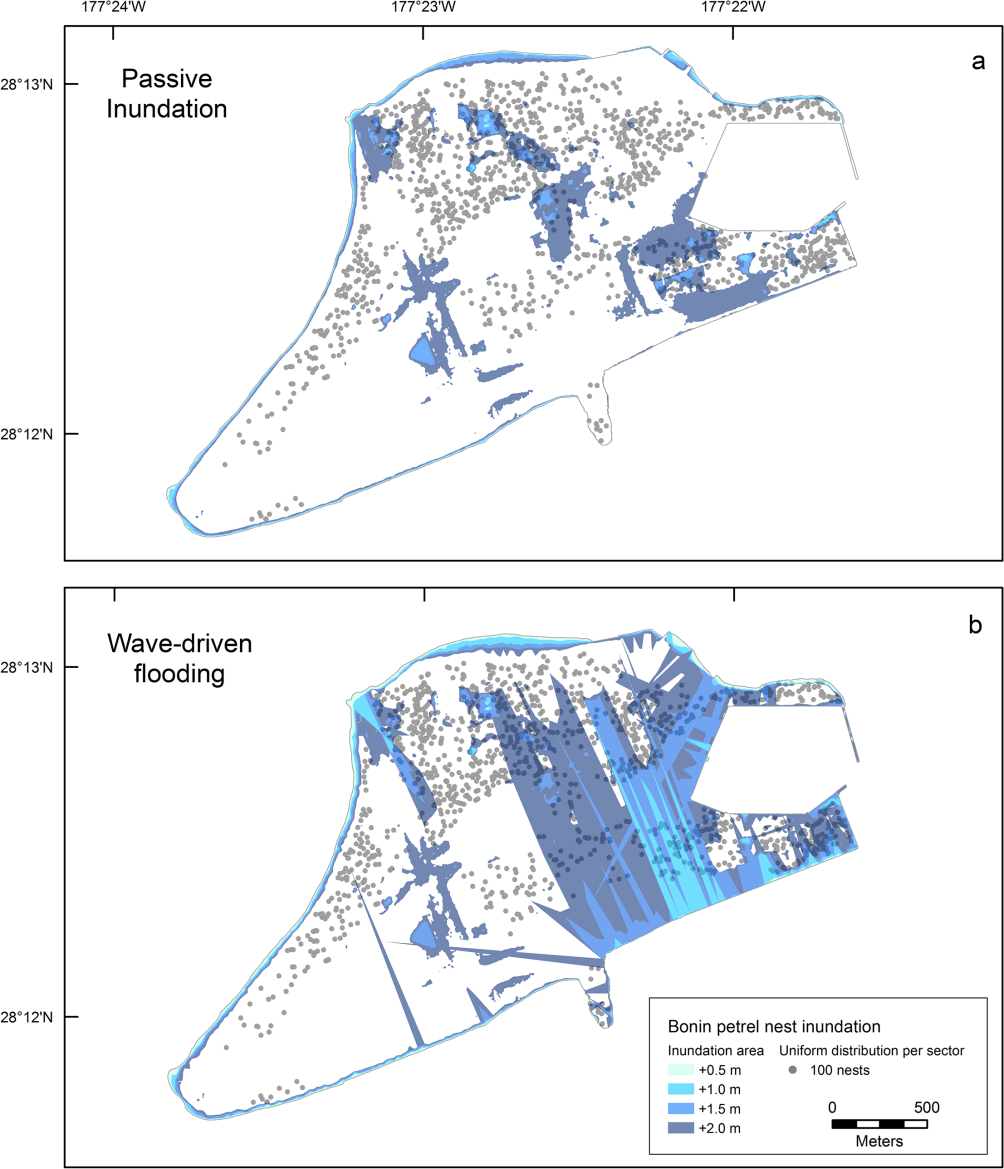
Projected Bonin petrel nests inundated at Sand Island, Midway Atoll for four different sea-level rise scenarios (+ 0.5, 1.0, 1.5, and 2.0 m) using passive (a) and wave-driven (b) models, including grounwater rise. Approximate nest locations assuming uniform distribution within colonies (known active nesting areas) stratified by estimated nesting density (see Methods for details). Inundation of Bonin petrel colonies at Eastern Island is not shown (refer to Table 5 for values). Bonin petrel nesting data was collected during 2008 (see [98]).

The Bonin petrel colony on Sand Island consisted of 129,733 breeding pairs (95% CI: 120,099–139,367); on Eastern Island approximately 200 pairs of Bonin petrels nested [98]. High-densities (1329 nests/ha) occurred in coastal and interior areas of Sand Island (Fig 5). Using the passive modeling approach, Bonin petrel inundation at Sand Island was 2% at +1.5 m SLR and 13% at +2.0 m SLR (Table 6). Using the wave-driven model at Sand Island, more than three times the nests were projected to be flooded (44%) at +2.0 m SLR. No Bonin petrel colony inundation occurred at +1.0 m passive SLR on Eastern Island and 67% of the colony area (ha) was inundated at +2.0 m passive SLR. With the wave-driven model at Eastern Island, we projected a loss of 13% of the nesting colony area at +1.0 m SLR, and nearly the entire colony at +1.5 m SLR (Table 6).

**Table 6:** Projected Bonin petrel nest inundation on Sand Island (a) and projected colony inundation on Eastern Island (b) for four sea-level rise scenarios and two inundation models, including groundwater rise. Nest distribution and abundance based on the 2008/2009 breeding season survey at Sand (129,534 nests [95% confidence interval: 120,099–139,367]) and Eastern (1.5 ha of colony area) islands [98].

** a**	**SLR Scenarios**							
	+0.5 m		+1.0 m		+1.5 m		+2.0 m	
Sand Island Model	No. Nests	Proportion	No. Nests	Proportion	No. Nests	Proportion	No. Nests	Proportion
Passive inundation	2	0.00	114	0.00	2,407	0.02	16,823	0.13
Wave-driven flooding	134	0.00	4,347	0.03	18,002	0.14	56,494	0.44
**b**	**SLR Scenarios**							
	+0.5 m		+1.0 m		+1.5 m		+2.0 m	
Eastern Island Model	Colony area (ha)	Proportion	Colony area (ha)	Proportion	Colony area (ha)	Proportion	Colony area (ha)	Proportion
Passive inundation	0.0	0.00	0.0	0.00	0.2	0.13	1.0	0.67
Wave-driven flooding	0.0	0.00	0.2	0.13	1.4	0.93	1.5	1.00

## Discussion

Atoll-wide inundation was projected to be less than 10% of the land area across all models for SLR scenarios less than +1.0 m, a reasonable planning target for the end of the 21^st^ Century [8, 15]. For the most vulnerable seabird populations identified, a threshold appeared in the passive inundation scenarios: at less than or equal to +1.5 m SLR, fewer than 3% of the nests atoll-wide were inundated for each of the Procellariiformes, but at +2.0 m SLR at least 13% of the Procellariiformes nests experienced inundation. A similar threshold in nest inundation appeared with the wave-driven models; however this flooding threshold appeared at + 1.0 or +1.5 m SLR. Thus, our models indicate that when storm waves are considered, seabird colonies will experience negative impacts from SLR much sooner than predicted by passive inundation models and at lower values of SLR. As such, passive inundation models that do not consider wave-driven flooding underestimate the impacts of SLR during storm events.

Not all species were vulnerable to rising sea levels in the same way. Laysan albatrosses are projected to be more vulnerable to rising groundwater than to wave-driven flooding. Conversely, black-footed albatrosses, which are concentrated around the coastal perimeter of islands, are more exposed to wave-driven flooding. Bonin petrels are equally vulnerable to rising groundwater and wave-driven flooding, although few nest on Eastern and Spit islands where inundation was more severe. Since albatrosses are ground nesters, we anticipate rising groundwater with SLR will decrease suitable nesting habitat availability over time, while wave-driven flooding will cause sudden nest failure and mortality. Adult Bonin petrel breeders may be even more sensitive to storm-wave overwash than the adult albatrosses because rapid flooding and burrow collapse causes below ground entrapment and drowning.

### Implications of SLR for Island Wildlife Populations

Approximately 66 Pacific islands are protected in vast U.S. Marine National Monuments, of these islands, 58 provide seabird nesting habitat and these rookeries are expected to be vulnerable to climate change [25, 32–36, 99]. Storm-wave flooding is projected to increase in frequency and magnitude due to climate change, and we expect this will alter both the availability of nesting habitat and seabird population dynamics. SLR is likely to reduce the carrying capacity of low-lying islands to support breeding colonies by decreasing land area available for nesting and by limiting vegetation regeneration as a result of frequent overwash events [100, 101]. Intermittent wave-driven flooding is more likely to drive colony failures and island population extirpations than gradual habitat losses resulting from passive inundation. Additive mortality, through sudden drowning of nests, chicks, and sometimes adult breeders may drive population declines (e.g., [102–105]), threatening species persistence.

Two sudden flooding events in 2011 illustrated the potential impacts of climate change and escalating SLR on seabird colonies of low-lying islands. A powerful winter storm and strong winds (in excess of 115 km per hour at Midway Atoll) occurred in February, followed by the Tohoku earthquake-generated tsunami [106, 107]. Losses across three Pacific atolls (Kure, Midway, and Laysan) were reported to be more than 250,000 Laysan albatross nests and 30,000 black-footed albatross nests [EF, unpublished data]. Additionally, adult albatrosses, Bonin petrels, great frigatebirds, red-footed boobies, black noddies, and white terns were observed drowned, entrapped by windblown sand, or killed by falling trees and uprooted shrubs [P. Leary, A. Kristof (USFWS), *personal communication*].

Wind and flooding exposure is low for species that do not breed during the winter storm season. For example, the February 2011 storm at Midway Atoll resulted in catastrophic nesting failure of albatrosses, yet wedge-tailed shearwaters and sooty terns were not breeding and avoided negative impacts. Likewise, only a portion of the breeding population of an asynchronously nesting species is affected by severe winter storm events (e.g. red-tailed tropicbirds). Additionally, in the NWHI, black noddies, white terns, sooty terns and red-footed boobies can re-lay if eggs or chicks are lost due to sudden inundation or other perturbations and therefore are less sensitive to a discrete catastrophic event. Additionally, white terns exhibit plasticity by co-occurring with introduced predators, recolonizing urban habitats, and persisting in highly modified environments, such as Honolulu, Hawai‘i [108].

In contrast, sudden and more frequent flooding associated with SLR will pose higher risks to Procellariiformes, with life history traits including singular nesting attempts, strong nest-site and mate fidelity, and rare dispersal to new breeding locations. Additionally, Procellariiformes may skip several years of breeding while establishing a new nest site or replacing a lost mate [92, 109, 110], thus as SLR occurs the impacts of lost reproduction will be magnified.

Regardless of dispersal propensity, dispersal opportunities to higher elevation sites without introduced mammalian predators are very limited [111]. Protected, predator-free seabird habitat in the western Pacific is largely restricted to the small low-lying islands within protected areas. As such, with rising sea levels and increased perturbations from storm wave overwash, contemporary colonial seabird habitat is likely to become increasingly unsuitable and may function as an ecological trap [48, 49, 112]. Decades of reproductive effort may be lost at colonies as low-lying islands continue to attract breeders via social attraction, despite chronic nest failure.

Under conditions of rapid environmental change, some intrinsic behavioral and life history traits may become maladaptive (i.e., evolutionary traps; [113]). In the case of long-lived and far-ranging Procellariiformes, their strong nest-site fidelity allows for long-term mate reunion and retention after distant pelagic migrations [56, 110, 114, 115]; however, strong philopatry to low-lying islands or coastal nest sites prone to sudden flooding may also be an evolutionary trap, threatening anthropogenically and geographically restricted species. For instance, several albatross colonies in the Hawaiian Islands persisted for more than a decade without offspring production. At present, returning adult Laysan albatrosses continue to attempt nesting at a historical colony near a U.S. Navy airfield on Kaua`i Island despite years (1988–2015) of complete reproductive failure due to egg removal to reduce bird airstrike hazards [116]. Additionally, highly philopatric Laysan and black-footed albatrosses continued to nest at eroding Trig Island (area 1.4 ha, mean elevation 0.5 m; [22]) in French Frigate Shoals (FFS) despite frequent overwash events driving chronic nest failure over the past decade. Likewise, albatrosses on the former Whale-Skate Island, FFS, returned to nest for years with no reproductive success before the island’s submersion in 1999 (EF, personal observation).

### Model Uncertainty

Multiple sources of epistemic uncertainty exist in our knowledge of physical and biological responses to climate change [117, 118]. The magnitude and rate of future SLR and storm events is subject to changing human and environmental conditions, such as carbon dioxide (CO2) emissions, temperature changes, ice sheet dynamics, glacial retreat, and oceanic heat uptake [8]. Currently, climate scientists have low confidence in projecting future tropical wind and storm patterns due to the coarse resolution of climate models [8]; thus, it is unknown if storm seasonality might change in the future.

Wave run-up sensitivity testing **i**ndicated low sensitivity of wave run-up heights to variations in model input. Modeled wave heights and periods were within 2% of the measured wave heights, with no difference in wave periods, while the standard deviation of slope was 2.18%, resulting in a mean error of wave run-up of 0.11 m, which is less than the RMSE of our DEM (0.39 m).

Our models depict inundation and flooding based on present day geomorphology, recent hindcast wave height data and seasonal oceanographic conditions. They do not include projecting changes due to sedimentation, erosion, and other coastal processes that would require extensive data on substrate, currents, hydrodynamic roughness, and use of more complex Delft 3D modules including FLOW (to model currents) and MOR (to address sediment transport and morphological changes) [74].

Tremendous uncertainty exists in seabirds’ physical and behavioral response to the rapid global changes. It is unknown if the breeding season of tropical seabird species might shift in response to climate driven changes in at-sea foraging conditions, as has been observed in some polar seabirds [119, 120]. Additionally, dispersal, survival, and reproductive rates of future more densely concentrated colonies are unknown, as is the maximum carrying capacity of most existing nesting islands.

### Management applications

Midway Atoll highlights the threat climate change poses for many Pacific island species [25]. Furthermore, our models demonstrate that negative impacts from sudden flooding events will occur long before seabird habitat is affected by passive inundation. Our findings suggest evaluation of conservation status for albatrosses and Bonin petrels may be warranted given projections of climate change driven sea-level rise (Table 1 [65–68]).

Opportunities for mitigation exist by restoring or creating habitat to increase the carrying capacity for seabirds in areas less vulnerable to inundation within their existing range [121]. High islands and mid-elevations are expected to be more ecologically stable to climate change [122], however, introduced predators and other anthropogenic threats to seabirds are pervasive there. In the main Hawaiian Islands, Kaho‘olawe (maximum elevation of 450 m. 11,700 ha) and Lehua islands (maximum elevation 213 m, 110 ha) are uninhabited by humans and could provide higher elevation refugia for nesting seabirds after the eradication of introduced predators [123, 124]. Additionally, other sites on populated higher Hawaiian Islands could provide more refugia from SLR and invasive predators with construction of mammal-proof fences [125, 126]. If seabirds can be protected from introduced mammalian predators, restoration of colonies to higher elevation islands represents an important conservation adaptation to rising sea levels and species currently restricted to low-lying islands.

## Supporting Information

S1 File File A, Number of Laysan (*Phoebastria immutabilis*) and Black-footed (*P. nigripes*) albatross nests counted. File B, Geographic delineations of areas where global positioning system (GPS) locations were collected at all Black-footed albatross nests. File C, Global positioning system (GPS) locations of Black-footed albatross nests. File D, Geographic delineations of Bonin Petrel (*Pterodroma hypoleuca*) nesting areas and their associated nesting densities. File E, Geographic delineations of passive and wave-driven sea-level rise inundation scenarios. (ZIP)
